# Measuring quality of life in mental health: Are we asking the right questions?

**DOI:** 10.1016/j.socscimed.2014.08.026

**Published:** 2014-11

**Authors:** Janice Connell, Alicia O'Cathain, John Brazier

**Affiliations:** a Health Services Research, School of Health and Related Research, University of Sheffield, Sheffield, UK; b Health Economics, School of Health and Related Research, University of Sheffield, Sheffield, UK

**Keywords:** UK, Quality of life, Mental health, Preference based measures, EQ-5D, SF-6D, Well-being, Recovery

## Abstract

Measuring quality-adjusted-life years using generic preference-based quality of life measures is common practice when evaluating health interventions. However, there are concerns that measures in common use, such as the EQ-5D and SF-6D, focus overly on physical health and therefore may not be appropriate for measuring quality of life for people with mental health problems. The aim of this research was to identify the domains of quality of life that are important to people with mental health problems in order to assess the content validity of these generic measures. Qualitative semi-structured interviews were conducted with 19 people, recruited from UK mental health services, with a broad range of mental health problems at varying levels of severity. This complemented a previous systematic review and thematic synthesis of qualitative studies on the same topic. Seven domains important to quality of life for people with mental health problems were identified: well-being and ill-being; relationships and a sense of belonging; activity; self-perception; autonomy, hope and hopelessness; and physical health. These were consistent with the systematic review, with the addition of physical health as a domain, and revealed a differing emphasis on the positive and negative aspects of quality of life according to the severity of the mental health problems. We conclude that the content of existing generic preference-based measures of health do not cover this domain space well. Additionally, because people may experience substantial improvements in their quality of life without registering on the positive end of a quality of life scale, it is important that the full spectrum of negative through to positive aspects of each domain are included in any quality of life measure.

## Highlights

• Seven domains important to quality of life were identified.
• Generic preference based QoL measures do not cover the life domains important in mental health.
• Depression has the greatest impact on quality of life.
• Measures should consider both what adds and takes away quality of life.
• There is a large overlap between the concept of quality of life and that of ‘personal recovery’.

## 1 Introduction

There has been a shift in mental health service policy from an emphasis on treatment focused on reducing symptoms, based on a narrow notion of pathology and illness, to a more holistic approach which takes into consideration well-being, recovery, social functioning, and quality of life (Hogan, 2003, Department of Health, 2011). A policy that more people attending mental health services will recover and have a good quality of life necessitates that appropriate outcome measures are in place. However few such measures are standardised and routinely collected across mental health services (Department of Health, 2011).

A review of eleven instruments for measuring quality of life for people with severe mental illness identified that the most commonly assessed domains are employment or work, health, leisure, living situation, and relationships (Van Nieuwenhuizen et al., 2011). However, concerns have been raised regarding the relative importance of the domains measured in such instruments (Dolan et al., 2008, Eack and Newhill, 2007).

At the same time there has also been a growing need for the economic evaluation of mental health services. This has resulted in an increased use of generic preference-based quality of life measures, such as EQ-5D (which measures mobility, self-care, usual activities, pain/discomfort and anxiety/depression) and the SF-6D (which measures physical functioning, role limitation, social functioning, pain, mental health and vitality). These measures are also used to estimate a score representing the health related quality of life. This is calculated on a scale where full health is one and states as bad as being dead is zero in order to calculate Quality Adjusted Life Years (QALYs; Dolan, 1997, Brazier et al., 2002). However, there is evidence that these generic measures may not be appropriate for people with the most severe mental health problems, particularly in psychosis (Papaionnou et al., 2011) and bi-polar disorder (Papaionnou et al., 2013, Hastrup et al., 2011) and there is limited evidence about their appropriateness for people with anxiety and personality disorder (Brazier et al., 2014). Some argue that these measures have been designed primarily for use within physical illness and thus place disproportionate importance on pain and disability rather than mental health (Saarni et al., 2010).

Quality of life measures have also been criticised for being generated from the perspective of mental health professionals rather than considering what individuals with mental health problems perceive to be important to their quality of life. It is recognised that the views of health service users should play a central role in the development and testing of patient reported outcome measures (US Department of Health and Human Services Food and Drug Administration, 2009).

As part of a wider study to explore the appropriateness of generic preference based measures for people with mental health problems (Brazier et al., 2014) we conducted a systematic review of qualitative research of the meaning of quality of life for people with mental health problems (Connell et al., 2012). We identified six domains of quality of life: well-being and ill-being; control, autonomy and choice; self-perception; belonging; activity; and hope and hopelessness. One limitation of the review was that available studies focused on quality of life of people with severe and enduring mental health problems, particularly schizophrenia. To complement the review we undertook primary research with people with severe and enduring mental health problems and mild-to moderate common mental health problems. This allowed us to explore the extent to which the review addressed important aspects of quality of life for those with severe mental health problems, given that most concerns have been expressed about the appropriateness of preference based measures in this group, and also address a gap in the current evidence base around the views of people with less severe problems.

## 2 Method

We undertook a qualitative study of face to face semi-structured interviews with current users of mental health services.

### 2.1 Recruitment

Participants were recruited from three National Health Service (NHS) mental health providers in a city in the north of England, UK. One primary care service provided psychological therapies for those with mild to moderate depression and anxiety (Improving Access to Psychological Therapies – IAPT). The other two specialist psychiatric services were for those with more severe problems (Community Mental Health Teams – CMHT), one working with individuals with severe and complex non-psychotic disorders (e.g. severe depression, post-traumatic stress disorder, personality disorder) and the other psychotic disorders (e.g. schizophrenia, bi-polar disorder). Recruitment was undertaken by service providers who applied wide inclusion criteria in order to capture as broad a range of mental health problems as possible. Exclusions included people experiencing acute episodes of their mental health condition, those not well enough to take part, where there was a known recent forensic history, and those who could not speak English or give consent. Further details on recruitment procedures can be found in Brazier et al. (2014). Approval for the research procedures was given by the local Research Ethics Committee, ref 10/H1308/11 and local NHS Research Governance, ref ZM03.

The services recruited 21 people to take part in the research and 17 were subsequently interviewed (two could not be contacted, one cancelled due to illness and one did not attend the arranged interview). Nine were recruited from the service for those with mild to moderate problems and eight from the two services for those with more severe problems. A further two participants diagnosed with schizophrenia were recruited by one of the participants subsequent to their own interview.

### 2.2 Interviews

All 19 participants were interviewed Sept–Nov 2010 by the first author, a mental health researcher with a background in behavioural sciences, mental health service evaluation and outcome measure development. The researcher had previous experience of interviewing people with mental health problems and had also undertaken training on qualitative methods at a leading centre of social research in the UK. The interviews were semi-structured with the use of a topic guide to ensure that a common set of questions were asked. The topic guide was based on the synthesis of the systematic review of qualitative research (Connell et al., 2012). The first part of the interview aimed to elicit what was important to quality of life from the perspective of the individual, without any prompts. They were asked general open ended questions about what affected their quality of life both from a positive and negative perspective, what they enjoyed and why, what they would most like to change, what helped, and what was stopping them doing what they wanted to do. Once their own perceptions had been thoroughly explored the interviewer introduced concepts from the systematic review (Connell et al., 2012) or were included in the EQ-5D or SF-36. These were raised only if they had not already been discussed in the interview and included questions about the relative importance or effect on their quality of life of relationships, support, stigma, work, leisure activities, mental health symptoms and relative affects, medication and side effects, physical health/pain, energy/motivation, self-esteem/confidence, mental health services/workers, finances.

All the interviews were tape recorded apart from one, at the request of the interviewee, where notes were taken; one further interview was recorded but accidently deleted so notes for this interview were made three days after the interview took place. The interviews lasted between 25 min and 1 h 50 min, averaging 1 h 16 min. Participants had the option of a home interview or at the University. Seven of the interviews took place in the participant's own home and the remainder in an interview room at the University.

### 2.3 Participants

Interviewees included 12 men and 7 women who had a broad range of mental health problems and levels of severity including schizophrenia, schizo-affective disorder, personality disorder, post-traumatic stress disorder (PTSD), mild to severe depression, anxiety, agoraphobia, eating disorder, and anger. Some presenting with mild to moderate problems had experienced more severe mental health problems in the past. With the exception of one, none of the participants were in paid employment at the time of the interview although most had worked at some time in the past or were currently working in a voluntary capacity. The majority (14/19) lived alone. Further information on the participants can be found in Table 1.

**Table 1:** Research participants.

	Gender	Age range	Relationship status	Recruited from	Problem/diagnosis disclosed by participant
1	F	40–49	Married	CMHT	Depression/eating disorder
2	M	20–29	Married	IAPT	Anxiety
3	M	40–49	Separated	IAPT	Depression/anger
4	M	40–49	Single	CMHT	Depression/anxiety
5	F	50–59	Married	CMHT	Depression/anxiety
6	M	60–69	Single	Other	Schizophrenia/depression
7	M	40–49	Married	CMHT	Depression
8	F	40–49	Widowed	CMHT	PTSD/depression/anxiety/agoraphobia
9	F	50–59	Divorced	IAPT	Depression
10	M	40–49	Divorced	IAPT	Anxiety/agoraphobia
11	F	30–39	Separated	IAPT	Depression/anxiety
12	F	30–39	Single	IAPT	Depression
13	F	30–39	Single	CMHT	Depression/personality disorder/social anxiety
14	M	30–39	Single	CMHT	Schizo-affective disorder
15	M	50–59	Single	IAPT	Depression
16	M	30–39	Single	CMHT	Schizophrenia/depression
17	M	50–59	Single	Other	Schizophrenia
18	M	60–69	Married	IAPT	Depression
19	M	40–49	Separated	IAPT	Depression

CMHT – Community Mental Health Team – severe mental health problems.

IAPT – Increasing Access to Psychological Therapies – mild to moderate mental health problems.

Other – Recruited via participant.

### 2.4 Analysis

The interview recordings were transcribed verbatim. Notes were used for two interviews. The data was analysed using framework analysis (Ritchie and Spencer, 1994). Framework analysis was used because it explicitly allows for both a priori themes and emergent themes. The themes identified in the review became the a priori themes of the framework (Connell et al., 2012). Next, each transcript was read for re-familiarisation and emergent themes or sub-themes identified. One new theme and a number of sub-themes emerged at this stage. Then interview text was coded and charted (verbatim or paraphrase) onto the thematic framework. This occurred with relative ease because of the fit between the a priori themes and the interview data. Nothing was considered irrelevant and all data was coded into a theme. All charted text was then indexed with the interview timings noted in the transcripts so that actual dialogue could be returned to, ensuring contextual accuracy. It was not unusual for text to be coded under more than one theme, often one descriptive and another conceptual; cross references between themes were noted. The final stage of framework is mapping, whereby the relationships between themes and sub-themes are considered. We were prepared to change the themes at this point to better reflect the content of the interviews. However, mapping did not result in a change to the original thematic framework. Finally, the content of each theme was compared with the content of the matching theme from the systematic review. At this stage it was apparent that although the main themes were the same, the content differed in terms of the wider spectrum of views of quality of life identified from our interviews and the identification of some new sub-themes.

To ensure the analysis was not unduly shaped by the systematic review (Connell et al., 2012), two independent researchers who had no previous knowledge of the systematic review were each given three different anonymous transcripts and asked to develop their own themes. Themes developed included background/history; mental health/mood; thoughts about self (e.g. confidence) and thoughts of others (e.g. stigma); pastimes/leisure; physical difficulties; relationships; values and treatments. Once the list of themes had been identified by the two researchers they were given the themes from the initial analysis of the interviews and asked if any themes were inappropriate or if new themes should be added. Although slightly different labels had been given to the two sets of themes (e.g. mental health/mood was similar to well/ill-being, and thoughts about self was similar to self-perception), the lead researcher (JC) and the two independent researchers concluded that it was not necessary to add new themes or change the original themes.

## 3 Findings

The importance of the quality of life domains identified in the systematic review was endorsed in this primary research. An additional domain of physical health was identified which appeared in the systematic review as a minor theme but was a major issue in this primary research. Within each theme we also identified differences of emphasis according to diagnosis and severity of problems. In particular, those with more severe problems at the interview spoke of things that took quality away, whereas those who had less severe problems at the time spoke of things that added quality. Finally, we identified additional sub-themes within some domains.

### 3.1 Well-being/ill-being

In the systematic review we found that the absence of ill-being was a particularly important aspect of quality of life, especially for those with severe or chronic mental health problems. This is a broad theme with various sub-themes important to quality of life including the feelings of distress caused by symptoms (for example the experience of psychosis/mania), depressed mood, problems with energy and motivation, and feelings of fear and anxiety. The detrimental effects of each of these aspects of psychopathology on quality of life were strongly supported by our interviews.

*I don't like to do nothing now because I have enough on my brain … I hear voices every day, I feel depressed, I get anxious … my energy level is downhill as well, and I'm getting anxiety unnecessarily, all the time anxiety disorder, unnecessarily, unhappiness and misery and everything … it stops me, it's sabotaging me in a way (16, CMHT, Schizophrenia)*

An additional aspect of ill-being not identified in the review was feelings of anger and frustration, which was present due to the inclusion of an interviewee receiving treatment for depression and anger:

*I get angry quite a lot and I'll just blow my top, I think I have calmed down a lot now … my life so far it has just been crap, just the violence most of my life and I don't want it, so I have got to change (3, IAPT, Depression/Anger)*

When interviewees were asked what would improve quality of life it tended to be the absence of the distressing negative feelings that took precedence rather than the presence of the positive aspects of well-being. Those participants who indicated co-morbidity of mental health problems (e.g. both psychosis and depression) were asked which of these feelings affected their quality of life the most; ‘depression’ was the unanimous response. They explained that this was due to the all-consuming nature of depression over which they felt they had no control. Whilst they could obtain some relief from anxiety or intrusive voices, depression was expressed as an ever present darkness that was difficult to escape or cope with.

*The depression is worse than the voices, because the voices sometimes, when you are with people, like now with you, I'm not hearing them, if you're doing serious matters, but I don't know how long that will be, with me not hearing voices, but the depression is always there … (16, CMHT, Schizophrenia)*

Due to the importance of the absence of feelings of distress for many participants, some interviewees, particularly those with schizophrenia and/or severe anxiety related problems, explained how they faced the dilemma of protecting their immediate well-being by opting for an easier, less stressful, but relatively boring life or risking relapse or exacerbation of their symptoms by engaging in difficult activities that could potentially improve their quality of life in the long term. The former approach of limiting their lives to obtain an immediate improvement in quality of life seemed to take priority, but not without some regret. Others, particularly those with less severe problems did continue to endeavour to resume activities they once enjoyed. There was often a discernible gap between what they would like in an ideal world and what they felt capable of at that time, and many described how they had to make compromises:

*what the psychiatrists advocate is stress, a relatively low stress environment, that's how I keep my mental health on an even-, you know, my mental health healthy as it were … part of me thinks that perhaps if I could do a job where even if it was just cleaning, and if I could, in a way, confront stress … I think to a certain extent, it would probably be a bit more healthy to have a bit more in my life (17, Other, Schizophrenia)*

Relief from distressing feelings was obtained through medication and holistic therapies but some participants with severe mental health problems engaged in coping strategies, such as the use of illegal drugs and alcohol, that again gave immediate relief but which they were aware could detrimentally affect their long term quality of life:

*if you try street drugs sometimes, speed, the voices turn into happy, happy voices all the way, and that's paradise … but please don't tell no-one [said quietly] because my worker is working hard with me to get me well and he doesn't encourage me to do street drugs, and I don't encourage myself to do street drugs (16, CMHT, Schizophrenia)*

The review and our interviews also identified aspects of well-being important to quality of life. These tended to be more about feeling healthy, peaceful, calm, relaxed, stable, safe and free from worry and demands rather than enjoyment and happiness (perhaps due to low expectations). For some, particularly for those with the most severe problems at the time of the interview, this was an ideal perceived to be almost beyond reach.

*I can remember when I used to be relaxed and chilled and not feeling like I do now, but I've felt like this for such a long time and anxious for such a long time, but I can remember what it was like not to feel like this, it's a marvelous feeling … I would like to be able to feel more relaxed (10, CMHT, Anxiety/panic attacks)*

On the other hand, those who were less distressed at the time of the interview spoke of experiencing positive well-being within certain areas of their lives, e.g. when pursuing leisure activities, or it was something they were actively striving towards.

*Interviewer: what makes you happy?*

*Interviewee: making sure that my wife is comfortable and happy, my son, my garden, it is just simple things really, there is no major thing that I can think of. (18, IAPT, Depression)*

### 3.2 Relationships and a sense of belonging

The review had identified that the concept of belonging, fitting with society, and the quality of relationships was important to quality of life. Similarly, our interviews revealed the importance of caring, loving and supportive relationships, companionship and camaraderie, together with acceptance and understanding from wider society. Detrimental to quality of life were critical and judgemental relationships, stigmatisation, rejection and lack of understanding by those close to them and wider society, all of which resulted in feelings of loneliness, isolation and detachment.

In contrast to the review, our interviewees felt that it was not only about how they fit with society but also about how society fit with them. There was a sense of feeling different and disconnected, rather than not ‘normal’ which had been a strong sub-theme in the review. Some expressed a dilemma about whether or not they wished to be part of a society that they felt had different values to their own. A sense of being detached from society was most expressed by those with severe depression and psychotic disorders.

*I have feelings of err not belonging to the human race, like I feel very-, it's not an outcast, I just don't feel a connection. I don't know how else to describe that, it's like being an alien, that is the only way I can describe it, and I know that that sounds weird but that is the only way that I can describe the feeling of it … I don't cope with most people [sighs] – my values and norms are very important to me and I know that everybody has not got the same ones (4, CMHT, Depression)*

Stigmatisation, the antithesis of a sense of belonging, was a strong theme in the review and whilst present in our interviews, was expressed as a problem primarily by those with schizophrenia and by the participant with gender identity problems. Stigmatisation was described as being less of a problem by those with anxiety and depression; however they were reticent about disclosing or talking about their problems with others for fear of being discriminated against or judged. Those with less severe depression and/or anxiety expressed feelings of being temporarily isolated from society rather than ultimately not belonging to it. In some instances withdrawal from family, friends or society was a necessary coping strategy. This had both a beneficial and detrimental effect on quality of life in that it made them feel less anxious and more able to cope but at the same time affected their self-esteem and confidence which in turn could increase feelings of depression.

*you just, I suppose, in some ways, you find ways of coping even though the ways of coping aren't very good, you find ways of dealing with life on a day to day basis, which basically means, you know, withdrawing from life and not having anything to do with people whatsoever … (it) is very important for me, not to isolate myself again, not to go back to being that person who doesn't want to have anything to do with people … (12, IAPT, Depression)*

Important to a sense of belonging was the experience of relationships with others. Consistent with the review, relationships had the capacity to both add and take quality away from life. Whatever their diagnosis, participants spoke of the benefits of having someone to talk to, feeling accepted and understood, experiencing love, care, affection and companionship, and having somebody they could rely on and trust. This could be from a partner, friends, family or health professional.

*Interviewer: what has brought about biggest change, what has helped the most?*

*Interviewee: having someone to talk to, by far, and understanding … and that is when I learned to love because I was accepted … because it makes you feel safe … somebody who doesn't judge and can accept you for who you are and who's not going to hurt you and take advantage of you (1, CMHT, Depression/Eating Disorder)*

Some of our interviewees, particularly those with severe and enduring mental health problems, revealed difficulties forming and maintaining the type of relationships they ultimately desired, often because of an abusive past. They spoke of feelings of rejection and being criticised, having difficulties with trust, and feeling anxious in social situations. This had a reciprocal detrimental effect on subjective well-being and self-perception.

*I alienate myself because I don't share. I have to take responsibility for that, but I can't-, without sharing they don't understand why I don't share, it's a bit of a vicious circle. But to have that trust to share, is quite difficult for me, but, of course it is, it is not going to be easy, it takes a long time for me to be friends or something with anybody really (4, CMHT, Depression)*

### 3.3 Activity

The findings related to activity from both the review and our interviews were very similar – that activity mostly had a positive effect on subjective and psychological well-being. Those with less severe problems at the time of the interview talked of the benefits of current activities whilst those with severe problems reported missing the activities they once enjoyed. Both leisure and work activity engendered a sense of belonging through social interaction and generated feelings of self-worth, pride and a sense of achievement. Interviewees reported wanting to do the normal activities that other people did. The more meaningful, purposeful and constructive the activity the better, rather than activity which merely ‘filled time’.

*I've got quite a lot to keep myself occupied erm I don't seem to be getting anywhere though at the moment I just occupy my time. I would like to be more involved and have some means of developing oneself and moving on and progressing instead of just filling time (6, Other, Schizophrenia)*

A few participants with severe depression reported finding little meaning or enjoyment in any pursuits, often due to difficulties with concentration or lack of energy and motivation. For some, though the activity may not provide enjoyment, it could still have the benefits of providing a structure and routine to the day, relieving boredom, keeping the mind active and, in particular, provided a distraction from their problems.

Also consistent with the review there were certain circumstances when activity could be detrimental to quality of life. This occurred when the activity, usually employment, felt beyond their capabilities. Reasons given for this were stress and anxiety related to pressure of work, problems with concentration and difficulties with social interaction. For those with psychotic disorders and PTSD, undue anxiety could exacerbate other mental health symptoms such as hearing voices and experiencing flashbacks. Quality of life was therefore about pursuing enjoyable and/or meaningful activity that did not inflame their mental health problems.

### 3.4 Self-perception

The concepts of self-efficacy, self-identity, self-stigma and self-esteem identified within the review were all revealed as having an effect on many aspects of our interviewees' lives. Irrespective of diagnosis, or severity of illness, interviewees reported how a lack of self-confidence stopped them doing the things they wanted to do, and being the person they wanted to be.

*Interviewer: of things that you have talked about that would improve your quality of life, what is the most important thing to you?*

*Interviewee: I think trying to improve my confidence and self-esteem because my self-esteem is quite low and erm it stops me from making friendships and having relationships with people (13, CMHT, Depression/Affective Personality Disorder)*

There was a recognition that if they were able to overcome the barriers that were stopping them doing the things they wanted to do, their quality of life would improve, but the fact that they found this so difficult had a further detrimental effect on self-esteem and feelings of self-worth.

*… it can be very frus-, I do get ever so upset with myself, which is a bit of a vicious circle because I am so annoyed with myself for being like this and I think, right, I'm going to push myself now and go for it, but it's much easier said than done (10, IAPT, Anxiety)*

Consistent with the review, a fragmented and incoherent sense of self and identity was reported by some interviewees, particularly those with psychosis where opposing thoughts and voices challenged the self.

*it's almost like this different personality of voices bouncing round your head, kind of, you know what you'd call your train of thought, … but yet there's other ones trying to, kind of, take over and become that train of thought and they've got their own voices and their own personalities and their own characteristics and you're like struggling to, kind of, stop them hijacking your brain, in a way, … … it's not a, kind of, epiphany or whatever, it's more a, a kind of, struggle for sanity and reality and who you are (14, CMHT, Schizo-affective disorder)*

For those with depression/anxiety this incongruous sense of self was expressed as a desire to return to the person they used to be, a person who was ‘*confident*’ (2,8,11), ‘*proud’ (2,18), ‘important’ (7); and ‘worthwhile’ (10)*

*all I want to do is try and-, somewhere along the line to try and get back to what I used to be like, where my confidence, I could build my confidence back up, and things like that because I find that now, I am now like forgetting things and I sort of like question myself (11, IAPT, Depression)*

### 3.5 Autonomy, control and choice

Consistent with the review, our interviews revealed the importance to quality of life of the related concepts of autonomy, control and choice. The review highlighted the complex juxtaposition between support and independence; the dilemma between needing support and valuing independence while not wishing to be too dependent. This same quandary was communicated by our interviewees. The need and desire for support was expressed primarily by those with severe mental health problems. Although they often spoke of an ultimate desire for independence there was an acknowledgement that support, and sometimes dependence, were necessary particularly during periods of illness when they were not in a position to help themselves. The level of desired dependence or independence therefore varied according to current circumstances and differed over time.

*I don't want to end up where like things get on top of me and I've got no help, or there is nobody there for me, because usually I'm the type of person who likes to do things myself, but like at this moment in time, I find that I can't do that because erm I don't know where to turn (11, IAPT, Depression)*

In contrast, those with less severe problems at the time of the interview were more likely to value and be actively working towards greater independence, particularly where physical health and mobility difficulties were the barrier to this. For them, being independent was important for dignity, pride and privacy whereas dependency resulted in feelings of guilt and of being a burden.

*Just to sort of do something on my own without like “oh well I've got to get out” or whatever, “will you come to bus stop with me”, or “got to ring a taxi”, just to be able to say ‘right I'm off’ … I am dependent on people (Interviewer: how does that make you feel?). Fed up sometimes, I mean don't get me wrong, I would go out with my daughter or my friend but that would be by choice … you know without her thinking “oh I can't do this cos I've got to make sure me mam's alright.” [9, IAPT, Depression]*

A sense of being in control precluded autonomy and independence. The review revealed that for people with psychosis related disorders the control they achieved through medication was an important aspect of quality of life. Feeling in control was linked to feelings of safety:

*If I take my medication then I'm more stable and I've got better control. It doesn't rule the voices out completely and doesn't stop the voices but err it does mean I'm more stable more able to cope (6, Other, Schizophrenia)*

A similar finding was shown for those with similar disorders where the reduction in heard voices and ability to distinguish reality from fantasy was important for both self and environmental control. At the negative end of the spectrum there was a deep fear of loss of control and its consequences, particularly for those who experienced anxiety, panic attacks, or psychotic episodes. This fear could mean that they did not feel they could leave the safety of their home.

*So I'm like frightened when I go out in case I have an episode, an ambulance gets called or the police, because when I have a flash back I can't explain myself because I am still in trauma (8,CMHT, PTSD)*

Gaining control often involved the development of coping strategies. Some of these strategies, such as social withdrawal, often provided short term relief and feelings of safety, but were recognised as being detrimental to quality of life in the long term. Thus trying to attain too much control was also identified as being detrimental to quality of life as it prevented taking the risks that could lead to a better quality of life. Our interviewees were very much aware of this but found it difficult to change and find the balance between the two.

*I seem a bit of a control freak, I want everything to be worked out before I decide to do a certain thing, you know, I want everything to be fairly straightforward and I mean, you can't, in a way, you can't live life like that, and yet I still want to live life like that, do you know what I mean? … it's about the stress, erm having faith or taking this, stepping out of your comfort zone, whatever you want to call it, yeah (17, Other, Schizophrenia)*

Also important to autonomy is the availability of choice and opportunity. As with the review, choice was particularly associated with having sufficient finances, which in turn was often linked with the availability of suitable employment. However, for the majority of our interviewees the opportunity of employment, or at least suitable employment that befitted their circumstances, values or expectations, was something they felt was denied, often due to perceived discrimination. Not having money meant they did not have the opportunity to pursue those things that could improve their lives in ways that were related to leisure activities, their environment or their physical and mental health care.

*I ended up going to the halfway house [name] at halfway homes they had lots of opportunities … they would encourage you and enable you to do these life things so I did driving, somebody else did fishing. … I don't think they have a training budget now which is a bit of shame because it opened my life to lots of things. I went on courses … I got the money for the driving, knowing that I could drive triggered off other things … so that's how my life got built by having guidance and opportunities really, and I think that's a big thing isn't it? (1 CMHT, Depression/Eating Disorder)*

### 3.6 Hope and hopelessness

The review showed how important a positive view of the future was to quality of life. This involved having goals and aspirations, and being involved in activities that were fulfilling and had meaning and purpose. These were necessary in order to instigate change and have hope for a better future. There were noticeable differences between our interviewees concerning how they viewed the future. This could be related to their expected level of achievement before they became ill, and whether or not they felt that their goals were attainable given time. The most negative outlook, a feeling of hopelessness and despair was reported by those for whom previous attempts at positive action to change their situation had failed, and coping mechanisms they previously drew upon no longer had an effect. As a result they felt ‘stuck’ and could not perceive how their situation might change in the future.

*I think it's hard for me to answer what is important to me because the things that I thought were important erm things like having a job erm a partner and family erm kind of seem out of reach … I tried to get back into paid work but it just felt really hard to manage, so I have kind of realised that having a job isn't going to make me happy like I thought it would. … it just makes it feel like torture because I feel like I can't win and I feel like I can't really, can't go on feeling like this anymore, (13, CMHT, Severe depression)*

A more positive outlook tended to be expressed by our interviewees referred via the primary care services who had less severe and/or relatively short lived mental health problems. They were more likely to talk about having goals and plans in place which they were actively working towards and which felt achievable. Some of these participants were able to describe the changes that took place as their mental health improved.

*The one thing that I used to do a lot is not think about the future, think a couple of days ahead and then not think about, you know, any further than that … now, one thing that's different from when I started going through this process is that I'm more willing to think further ahead, I'm more willing to say, well in a year's time I'd like to be at this place, you know. (12, IAPT, Depression)*

### 3.7 Physical health

Whereas physical health problems did not feature strongly in the review, the majority of our interviewees reported physical health problems which impacted on their quality of life. There was a complex interaction between mental and physical health. For some, physical health problems contributed to a deterioration in their mental health.

*I've got diabetes, I've got high blood pressure, I've got arthritis, I've got angina, I'm on too many medications. I don't know what they are all for and it's just this last 6 months it has just been getting me down. (3, IAPT, Depression/Anger)*

Conversely, mental health problems also had a direct negative impact on physical health. Difficulties with sleep patterns and not eating properly took a toll on physical health. There were also physical side effects to medication, particularly those for psychosis including physical feelings of restlessness and agitation. Our interviewees also felt that their mental health problems caused or intensified aches and pains.

*… it feels physical as well as mental … my body aches and like I think I just become really tense and that is what makes my body ache and I feel like erm I feel like my chest is being crushed and erm I can't breath and things like that and erm I just want to be asleep all the time to escape but I can't sleep (13, CMHT, Depression/Affective Personality Disorder)*

*I think it is because I am that stressed out, and that hyped up, this is where the problem is, and when things are on my mind I don't sleep at all. It has affected my health and everything and I think that's why I have got these other issues now where my body is just in pain all the time … and I think it is through everything what I am going through, it is causing me to have all these problems (12, IAPT, Depression)*

Participants were asked which affected their quality of life the most, their physical or mental health problems. Most found this difficult to answer and described how they interacted with each other and how the presence of both made life particularly difficult to cope with.

## 4 Discussion

The aim of this research was to identify the domains of quality of life that are important to people with mental health problems. This was achieved by conducting qualitative semi-structured interviews with people with mental health problems which complemented a systematic review of qualitative research by broadening the range and severity of mental health problems examined. Seven domains important to quality of life for people with mental health problems were identified: well-being and ill-being; physical health; control, autonomy and choice; self-perception; relationships and belonging; activity; hope and hopelessness. Despite widening the types and severity of mental health problems studied, we found our interview data confirmed themes from the systematic review with the addition of physical health as a major theme. However the interviews highlighted how these domains differed by severity, chronicity and diagnosis. With some exceptions, those with severe and distressing difficulties (at the time of the interview) were more likely to talk about losses – what took quality away from life, whereas those with moderate or relatively short lived problems spoke more of the things that added quality to life.

The dichotomy between what adds and takes away from quality of life was particularly evident in our first theme ‘well-being and ill-being’. When interviewees were asked what would improve quality of life, those who were most distressed, spoke predominantly of the importance of the absence of negative feelings of ill-being (depression, anxiety, stress). When positive aspects of well-being were identified, they concerned a need to feel calm, contented and relaxed which predominated over feelings of elation or happiness. Furthermore, our interviewees stated unequivocally that depression had the most profound impact on quality of life when compared with their other mental health problems such as anxiety or psychotic symptoms. This is consistent with research that indicates that the severity of depression symptoms has been found to be most consistently and strongly related to subjective quality of life (Hansson, 2006). This suggests that anxiety and depression should be treated as separate items rather than combined as they are currently in the EQ-5D and that depression should be given a greater weighting.

Similar differences of emphasis and intensity relating to diagnosis and severity were found within the other themes. For example, within relationships and belonging, those with severe depression and psychosis spoke of being stigmatised and alienated from society, and having problems forming relationships whereas those with less severe problems experienced problems within specific relationships rather than relationships as a whole. On a positive note, feeling loved and cared for was important for quality of life. Being active was confirmed as being important to quality of life for all participants regardless of diagnosis or severity. However for those with severe problems activity could be detrimental to quality of life when it was deemed beyond the capabilities of the individual and resulted in an increase in symptoms. Additionally, feelings of hopelessness and despair were felt by some participants whereas others with less distressing or chronic difficulties were more optimistic.

A further aspect of the measurement of quality of life revealed in our research is the dilemma experienced between needs and aspirations. This can most clearly be seen within the theme of autonomy; there was often an aspiration for greater independence but an acknowledgement of a need and desire for support, and sometimes dependency, particularly during severe illness episodes when they did not have the cognitive abilities to deal with day to day problems. Similarly, there was a desire to work and the benefits this bestowed were well recognised, but the fear of exacerbating symptoms proved prohibitive. Additionally, social relationships were desired but withdrawing from society gave them the ability to cope. Our interviewees expressed how they engaged in negative coping strategies, such as avoidance, which made their life easier, but at the same time stopped them doing those things that might ultimately enhance their quality of life. It was therefore difficult for them to decide which was more important to quality of life – a restricted life, free from anxiety and stress, or a fuller life which involved anxiety and stress but which comes with a risk of exacerbating other aspects of their mental illness. The complexities outlined above may in some way be addressed by considering capability theory (Verkerk et al., 2001, Sen, 1993) and response shift (Sprangers et al., 1999). Capability theory considers the ability of a person to achieve that which is valued given available resources and opportunity and also whether the person chooses to benefit from them (Sen, 1993). Response shift, on the other hand, examines the changing values and re-conceptualisation of quality of life that occur as people adapt to chronic health problems (Sprangers et al., 1999). A possible example of response shift in our research was the desire for an absence of depression rather than the presence of happiness. However, care should be taken when making judgements as such adaptations and coping mechanisms can result in a true improvement in quality of life (Ubel et al., 2010). A response shift may be what one is looking for in outcome measurement – that the person perceives themselves as having a good quality of life despite their illness.

The findings of this study indicate that generic preference based measures (EQ-5D and SF-6D) do not include many aspects of quality of life valued by those with mental health problems. The themes of control, autonomy and choice; self-perception; belonging; and hope/hopelessness, all considered as important by those with mental health problems, are not addressed within the EQ-5D or the SF6-D. The single dimensions of anxiety and depression in the EQ-5D and mental health in the SF-6D do not cover the full spectrum from ill-being through to well-being nor does the EQ-5D consider the greater impact of depression over anxiety; in both measures the activity questions do not allow for the finding that some activity can have a negative as well as a positive impact.

One of the key criticisms of the EQ-5D and SF-36, from which the SF-6D is derived, is that they have been designed by researchers with little or no input from people with the relevant health problems. A recent example of a generic instrument developed using the general population is the ICECAP-A (Al-Janabi et al., 2012). The resultant classification system is less focused on physical health and functioning, and instead takes a higher level and broader view of the constituents of quality of life. It covers five dimensions of health: feeling settled and secure; love, friendship and support; being independent; achievement and progress; and enjoyment and pleasure. The ICECAP-A has similarities to our themes and is potentially more relevant to the mental health population. However, probably because the measure was developed using interviews with the general public rather than those who had experience of mental health problems, it only utilises the positive end of the scale. The negative end of the spectrum would need to be added for the measure to be useful for measuring quality of life in people with mental health problems.

There have also been recent advances in the measurement of ‘personal recovery’ a concept born out of service user representation and defined as ‘a way of living a satisfying, hopeful, and contributing life even with limitations caused by illness’ (Anthony, 1993). A recent systematic review of the literature, published after our own review had been completed, identified five recovery processes comprising ‘connectedness’, ‘hope’, ‘identity’, ‘meaning in life’ and ‘empowerment’ (CHIME; Leamy et al., 2011) which are very similar to our own final themes. Again, there is a tendency towards the positive end of the spectrum, and the concept does not include items relating to well/ill-being, both presumably due to its grounding in positive psychology and the rejection of an emphasis on symptoms and morbidity (Shepherd et al., 2008).

A major concern with using normative QOL or well-being measures, particularly those with a positive orientation, in severely mentally ill population is that floor effects (those with the worst scores can deteriorate further) are frequently encountered (Lehman, 1996). This is likely to result in the measure not being sensitive to deterioration or small positive improvements for those with severe and/or acute problems presenting at specialist mental health services. For instance, the absence of happiness may not be sensitive to changes at the severe levels of depression. Similarly the absence of a loving caring relationship may not incorporate the effects of stigma and rejection especially as negative social exchanges have been shown to have a greater impact on quality of life than positive ones (Newsom et al., 2005). Further, when examining the concept of hope and hopelessness, an item such as ‘I can achieve and progress in all aspects of my life’ found in ICECAP-A may not capture the very severe levels of hopelessness or desperation reported in some interviews where the interviewee felt they could no longer continue and had considered ending their lives.

This is not to say that the promotion of well-being and positive psychology within mental health services isn't important, however it is advised that both ends of the spectrum be incorporated so that a measure is sensitive to important changes and reflects the difficult journey to recovery of quality of life no matter the initial severity of the mental health problem. It is therefore necessary to examine empirically whether a single positive, or negative, item adequately measures the full extent of severity of any mental health condition and to develop a measure accordingly.

### 4.1 Strengths and limitations

The strength of this study was that it was undertaken in complement to a systematic review of qualitative research, thus building on previous evidence by expanding the types and severity of mental health problems included. To a large extent we achieved this, with interviews being undertaken with people recruited from primary and secondary services catering to the needs of people with severe and enduring problems and mild to moderate anxiety and depression.

Our intention had been to interview between 24 and 30 people because the interview study was undertaken in complement to a systematic review of qualitative research, rather than as a standalone study. Recruitment stopped at 19 interviewees partly due to data saturation and partly due to our inability to include some diagnoses in our sample, despite attempts to do so. With 19 interviews, we felt that we had achieved a wide spectrum of severity of mental health problems and that there was considerable repetition of themes which suggested data saturation for the diagnoses included. However, some diagnoses were missing from our sample and people with these diagnoses may have had different views of quality of life from those included in our study. We were unable to recruit women with psychosis related disorders, or people with Obsessive Compulsive Disorder and only one person with Personality Disorder. Our sample was primarily users of mental health services rather than a wider population of people with mental health problems and this may have resulted in an over-emphasis on the negative rather than positive aspects of quality of life. These interviews could be challenging to undertake but the interviewer (JC) had considerable experience of working with people with a spectrum of mental health problems in past research studies. At times some interviewees became upset and this may have affected the content of the interview and the subject matter discussed. The interviewer (JC) was aware during the interviews that, because of its personal nature, questions about close and sexual relationships were sometimes difficult to raise, resulting in this subject area not being fully explored.

A further possible limitation might be the presence of the systematic review and the influence this had on our analysis. We were aware of the possibility that this might blind us to aspects of our data, or unduly shape our analysis, and sought both to actively seek out differences and to undertake an external review of our analysis to minimise this.

### 4.2 Conclusions

The aim of this research was to establish the domains of quality of life that are important to people with mental health problems. Our interviews with people with mental health problems, together with results from our previous synthesis of qualitative research, have together identified seven domains of quality of life: well-being and ill-being; physical health; control, autonomy and choice; self-perception; relationships and belonging; activity; and hope and hopelessness. These domains should form the basis of the future development of measures to address quality of life from the perspective of people with mental health problems. Existing generic quality of life measures such as the EQ-5D and SF-6D do not address many of the domains. Such a measure should also consider the relative effect of depression and the importance of the absence of negative experiences as well as presence of positive experiences on quality of life. People enter mental health services with varying degrees of distress and chronicity, and as our research shows, the recovery of quality of life can be a long and difficult journey. Because people may experience substantial improvements in their quality of life without registering on the positive end of a quality of life scale, we argue that it is important that the full spectrum of negative through to positive aspects of each domain are included within quality of life measures; the need for positive and negative items in each theme should be explored empirically. Together with the findings from the review of the quality of life and recovery literatures, this provides the basis for informing the development of a more comprehensive outcome measure for use in mental health populations.

## References

[bib1] Al-Janabi H., Flynn T.N., Coast J. (2012). Development of a self-report measure of capability wellbeing for adults: the ICECAP-A. Qual. Life Res..

[bib2] Anthony W.A. (1993). Recovery from mental illness: the guiding vision of the mental health service system in the 1990s. Psychosoc. Rehabil. J..

[bib3] Brazier J., Roberts J., Deverill M. (2002). The estimation of a preference-based measure of health from the SF-36. J. Health Econ..

[bib4] Brazier J.E., Connell J., Papaioannou D., Mukuria C., Mulhern B., O'Cathain A., Barkham M., Knapp M., Byford S., Gilbody S., Parry G. (2014). A systematic review, psychometric analysis and qualitative assessment of generic preference-based measures of health in mental health populations and the estimation of mapping functions from widely used specific measures. Health Technol. Assess..

[bib5] Connell J., Brazier J.E., O'Cathain A., Lloyd-Jones M., Paisley S. (2012). Quality of life of people with mental health problems: a synthesis of qualitative research. Health Qual. Life Outcomes.

[bib6] Department of Health (2011).

[bib7] Dolan P. (1997). Modelling valuations for EuroQol health states. Med. Care.

[bib8] Dolan P., Peasgood T., White M. (2008). Do we really know what makes us happy? A review of the economic literature on the factors associated with subjective well-being. J. Econ. Psychol..

[bib9] Eack S.M., Newhill C.E. (2007). Psychiatric symptoms and quality of life in schizophrenia: a meta-analysis. Schizophr. Bull..

[bib11] Hansson L. (2006). Determinants of quality of life in people with severe mental illness. Acta Psychiatr. Scand..

[bib12] Hastrup L.H., Nordentoft M., HjorthÍj C., Dorte Gyrd-Hansen D. (2011). Does the EQ-5D measure quality of life in schizophrenia?. J. Ment. Health Policy Econ..

[bib13] Hogan M. (2003). New freedom commission on mental health, achieving the promise: transforming mental health care in America. Psychiatr. Serv..

[bib14] Leamy M., Bird V., Le Boutillier C., Wiliams J., Slade M. (2011). A conceptual framework for personal recovery in mental health: systematic review and narrative synthesis. Br. J. Psychiatry.

[bib15] Lehman A.F. (1996). Measures of quality of life among persons with severe and persistent mental disorders. Soc. Psychiatry Psychiatr. Epidemiol..

[bib16] Newsom J.T., Rook K.S., Nishishiba M., Sorkin D.H., Mahan T.L. (2005). Understanding the relative importance of positive and negative social exchanges: examining specific domains and appraisals. J. Gerontol. Ser. B Psychol. Sci. Soc. Sci..

[bib17] Papaionnou D., Brazier J.E., Parry G. (2013). How to measure quality of life for cost effectiveness analyses in personality disorders? A systematic review. J. Personal. Disord..

[bib18] Papaionnou D., Brazier J., Parry G. (2011). How valid and responsive are generic health status measures, such as the EQ-5D and SF-36, in schizophrenia? A systematic review. Value Health.

[bib19] Ritchie J., Spencer L., Bryman A., Burgess R.G. (1994). Analysing Qualitative Data.

[bib21] Saarni S., Viertio D., Perala J., Koskinen S., Lonqvist J., Suvisaari J. (2010). Quality of life of people with schizophrenia, bipolar disorder and other psychotic disorders. Br. J. Psychiatry.

[bib22] Sen A.K., Nussbaum M.C., Sen A.K. (1993). The Quality of Life.

[bib23] Shepherd G., Boardman J., Slade M. (2008).

[bib24] Sprangers M.A.G., Carolyn E., Schwartz C.E. (1999). Integrating response shift into health-related quality of life research: a theoretical model. Soc. Sci. Med..

[bib25] Ubel P.A., Peeters Y., Smith D. (2010). Abandoning the language of ‘response shift’: a plea for conceptual clarity in distinguishing scale recalibration from true changes in quality of life. Qual. Life Res..

[bib26] US Department of Health and Human Services Food and Drug Administration, Center for Drug Evaluation and Research (CDER), Center for Biologics Evaluation and Research (CBER), Center for Devices and Radiological Health (CDRH) (2009).

[bib27] Van Nieuwenhuizen C., Schene A.H., Boevink W.A., Wolf J.R.L.M. (2011). Measuring the quality of life of clients with severe mental illness: a review of instruments. Psychiatr. Rehabil. J..

[bib28] Verkerk M.A., Busschbach J.J.V., Karssing E.D. (2001). Health-related quality of life research and the capability approach of Amartya Sen. Qual. Life Res..

